# Self-contained intraoperative surface acquisition on a mixed-reality headset: a comparison of sensor-based and learning-based reconstruction

**DOI:** 10.21203/rs.3.rs-10437300/v1

**Published:** 2026-07-24

**Authors:** Bowen Xiang, Michael I. Miga

**Affiliations:** 1 Department of Biomedical Engineering, Vanderbilt University, Nashville, TN, USA; 2 Vanderbilt Institute for Surgery and Engineering (VISE), Vanderbilt University, Nashville, TN, USA

**Keywords:** Mixed reality, Surgical navigation, Intraoperative surface acquisition, Time-of-flight depth sensing, Multi-view depth estimation, Image-guided surgery

## Abstract

**Purpose:**

Mixed-reality (MR) surgical navigation requires an intraoperatively measured organ surface for deformable image-to-physical registration, yet commercial headsets often rely on manual stylus digitization or external sensors. This work evaluates whether Magic Leap 2 (ML2) onboard sensors can provide this surface without external hardware, and whether its depth sensor or an RGB learning-based reconstruction is more accurate.

**Methods:**

Two paradigms were developed from identical ML2 input in a common SLAM-tracked frame. The first used the onboard short-range indirect time-of-flight (iToF) stream. The second recovered geometry from an RGB sweep using Depth Anything 3, with metric scale anchored by SLAM-tracked camera centers. Both were evaluated against optically tracked stylus ground truth on opaque plaster and translucent silicone liver phantoms, a surface-treated breast phantom, and an ex vivo porcine liver, with five runs per specimen. Sensor-based feasibility was also demonstrated in vivo on an anaesthetized pig.

**Results:**

ML2 world-origin drift remained below 2 mm median across four perturbations, and the iToF sensor achieved approximately 2 mm absolute accuracy over 0.30–0.70 m. Accuracy depended on surface optics. The sensor-based method was more accurate on opaque plaster livers (RMSE 2.6 and 3.1 mm versus 4.7 and 5.0 mm), whereas the learning-based method was more accurate on translucent silicone livers (4.3 and 4.6 mm versus 4.8 and 5.3 mm). The sensor-based method also performed better on the sunscreen-coated breast phantom (2.9 versus 3.9 mm) and retained a modest advantage on the ex vivo porcine liver (4.2 versus 4.8 mm). In vivo, the sensor cloud showed a mean residual of 3.4 mm to its fitted surface.

**Conclusion:**

A commercial MR headset can acquire intraoperative organ surfaces using onboard sensors alone. Sensor-based reconstruction is preferred when active depth returns are reliable, whereas RGB learning-based reconstruction is more robust to optical failure modes but requires adequate multi-view coverage.

## 1 Introduction

### 1.1 Clinical background and motivation

Mixed reality (MR) head-mounted displays can overlay patient-specific preoperative models onto the surgeon’s view, improving intraoperative spatial awareness. For this to be quantitatively meaningful the model must be registered to the intraoperative anatomy, and in soft-tissue surgery this is not a one-time rigid step: organs such as the liver and breast deform substantially between imaging and operative presentation, so guidance depends on a deformable image-to-physical registration driven by an intraoperatively measured organ surface [1]. The denser and more accurate that surface, the better the deformation correction it supports.

Yet this surface is not natively acquired by commercial MR devices; current practice relies on manual digitization or auxiliary sensing devices. In liver surgery, it is not uncommon for this process to be achieved by swabbing point-by-point with a tracked stylus [2] the exposed liver. This process is slow, operator-dependent, somewhat cumbersome, and produces sparse data. In breast-conserving surgery an external stereo-camera system has been proposed to capture this data [1], but expressing that surface in the navigation frame requires an in-room calibration once the setup is fixed and the device is usually mounted separately, thus having limited integration. More generally, every external device must also be positioned, draped, kept sterile, and held in unobstructed line of sight, increasing the operating-room footprint and points of failure, and its fixed vantage is readily occluded by the surgeon’s hands; a headset-borne sensor moves with the surgeon’s viewpoint, so wherever the organ is visible to the operator it is visible to the sensor.

This motivates acquiring the surface directly from sensors already built into the MR headset. A recent comparison of the three leading commercial MR HMDs (HoloLens 2, Magic Leap 2, Apple Vision Pro) for a high-precision navigation task identified the Magic Leap 2 (ML2) as most effective overall, chiefly for its stable tracking [3]. That stability is essential: a stable SLAM-tracked world frame lets the views from a sweep be fused into one surface directly, without a separate multi-view registration step. We develop on the ML2, though the methodology is device-agnostic, as contemporary HMDs share an RGB camera, an active depth sensor, and inside-out SLAM. A challenge for any such device is that active depth sensing is biased by subsurface scattering on translucent, perfused tissue which motivates the two complementary paradigms compared here.

### 1.2 Related work

Prior work on MR-guided navigation, particularly for the liver, can be grouped by data processing stage including intraoperative segmentation and tracking, surface acquisition, or model registration. A common finding is that relying on the headset’s onboard depth sensor as the measurement source is insufficient. Prior groups have responded in one of two ways: either moving the problem into the image domain, or adding an external depth device to the setup.

On segmentation and tracking, Khajarian et al. [4] combined a domain-specific RGB-D network, the Segment Anything Model, and a video object-segmentation tracker with scene-aware re-prompting to achieve real-time liver segmentation on intraoperative HoloLens 2 data. Their work concerns segmentation accuracy and temporal resolution rather than the measurement fidelity of the reconstructed surface, which is never assessed against an independent ground truth.

A second approach sidesteps the onboard depth sensor by adding an external RGB-D camera [5]. The group’s reason was based on the finding that the onboard depth sensing was insufficient for high-precision use, and instead, used the HoloLens 2 only for display while delegating depth to an external Intel RealSense camera. Aliani et al. [6] similarly textured CBCT-derived head models with an Intel D435 camera registered through a ChArUco board, using the HoloLens 2 purely for visualization. Both had the effect of reintroducing the external hardware and some requiring fiducial calibration which is counter to our goal of creating a self-contained guidance headset.

The most direct evidence on the onboard sensor comes from Kerkhof et al. [7], who characterized the HoloLens 2 depth sensor in its Articulated Hand Tracking and Long-Throw modes and built a depth-based image-to-patient registration around it. They reported a consistent depth overestimation of several millimeters and an overall registration accuracy of roughly one to two centimeters, concluding the onboard sensor is not yet accurate enough for many surgical applications without further calibration, and leaving open whether the depth channel is even the best way to recover surface geometry on such hardware.

In sum, prior work has not established whether the depth sensor built into a commercial MR headset is itself adequate as the metric source for intraoperative surface acquisition, nor whether a learning-based reconstruction from the headset’s RGB stream alone could match or surpass it on the same device. These are the questions our work addresses.

### 1.3 Contributions

We develop and evaluate a self-contained, organ-agnostic pipeline for intraoperative surface acquisition on the Magic Leap 2, using only the headset’s onboard sensors and expressing every captured view in a single SLAM-tracked world frame so that multi-view aggregation requires no inter-view registration. The proposed data processing framework makes no organ-specific assumptions; we validate it across a phantom set spanning distinct surface-optical characteristics, including both liver and breast geometries:

1. A device-native, self-contained surface-acquisition workflow on a commercial MR headset requiring no external sensors or tracking hardware, together with a characterization of platform suitability: hologram (world-origin) stability under clinically realistic perturbations and measurement characterization of the onboard short-range iToF depth sensor.
2. Two reconstruction approaches developed on identical input: a sensor-based reconstruction that reads geometry directly from the onboard iToF stream, and a learning-based reconstruction that discards the depth channel and recovers geometry from the RGB sweep alone using a multi-view geometric foundation model (Depth Anything 3 [8]), anchored to a reference scale provided by the SLAM-tracked camera centers.
3. A direct, head-to-head evaluation of the two approaches against an independent, optically-tracked ground truth, stratified by tissue type (opaque versus translucent phantom), which makes explicit the trade-off between the absolute positional accuracy of the sensor-based surface and the local surface fidelity of the learning-based surface.

## 2 Methods

This section develops and compares two approaches for acquiring an intraoperative measurement of the three-dimensional organ surface on the Magic Leap 2 (ML2). The approaches are tested on liver and breast phantoms to establish their suitability of use for image-to-physical registration with 3D models derived from previous CT imaging of the phantoms. Both aggregate multiple views captured as the operator translates the headset across the field, and both express the final surface in a single ML2 SLAM-tracked world frame *F*_ML2_; they differ only in how per-view geometry is obtained. Method 1 reads three-dimensional structure directly from the onboard short-range indirect time-of-flight (iToF) depth sensor. Method 2 discards the iToF channel and recovers geometry solely from the RGB stream using a learned multi-view geometric foundation model (Depth Anything 3). The central question is which yields the more accurate surface against an independent, optically-tracked ground truth (an NDI Polaris Vega optical tracker with a pivot-calibrated stylus; acquisition detailed in Sec. 2.5).

Because both methods rely on the stability of *F*_ML2_ across a sweep, we first verify the tracked world origin is stable under operator-induced perturbations (Sec. 2.1). We then calibrate and characterize the two shared sensors, the RGB camera and iToF sensor (Sec. 2.2), and describe the concurrent RGB–D acquisition and SAM 2 segmentation producing a per-scan organ-only point cloud and mask shared by both methods (Sec. 2.3). The two reconstructions follow in Sec. 2.4 and the ground-truth comparison in Sec. 2.5.

### 2.1 Hologram stability evaluation

Aggregating views into a single cloud is meaningful only if the per-frame extrinsic from the ML2’s onboard SLAM stays locked to a fixed physical frame *F*_ML2_ throughout a sweep; drift would be indistinguishable from a per-view position bias and propagate into either reconstruction. We therefore quantify world-origin stability under operator-induced perturbations, following the HoloLens 2 protocol of Xiang et al. [2] with the ML2 substituted as host.

The setup comprised the ML2, an NDI Polaris Vega optical tracker (Northern Digital Inc., Waterloo, ON, Canada), and a pivot-calibrated stylus. A holographic cube rendered through the Magic Leap SDK in Unity and anchored to a persistent spatial anchor 10 cm above a tabletop served as the reference whose drift was measured: the operator digitized its four top vertices in a consistent sequence with the tracked stylus, each recorded through the PLUS toolkit integrated with 3D Slicer.

Four perturbation conditions, identical to [2] for comparability with the prior HoloLens 2 study, were evaluated: (1) walking 7 m from the hologram and back; (2) rapid head movement with sudden lateral rotations; (3) sensor occlusion until tracking was lost and re-localized; and (4) inserting a new object into the mapped region for 60 s to force a spatial-mesh update. Each used N = 40 trials, with vertices digitized once before and once after the disruption. Per-trial drift was the mean Euclidean displacement between corresponding vertices, recorded in the optical-tracker frame *F*_OT_, independent of the headset’s own coordinate system. The Polaris Vega XT contributes ≈ 0.64 mm RMS uncertainty per vertex, the floor of detectable drift. Distributions are reported by median, interquartile range, and full range.

### 2.2 Depth-sensor characterization

The sensor-based reconstruction is limited by the measurement fidelity of the iToF sensor, whose absolute accuracy and temporal stability set its noise floor; we characterize it before acquisition. The RGB camera’s intrinsics, which govern the image-to-3D projection in Method 1, are likewise recovered by offline calibration.

The iToF sensor is accessed through the same OpenXR extension in its short-range stream, specified over 0.2–0.7 m [9], matching the headset-to-organ standoff of typical surgery. The depth image, its per-pixel quality-flag buffer, and the calibrated pinhole intrinsics are queried from sensor metadata, so any factory recalibration propagates downstream. Pixels marked invalid by the quality flags are removed before any geometric use. The short-range iToF stream reports the radial distance *r* from the optical center along each ray rather than the camera-frame depth *Z*. Substituting *r* for *Z* would make a flat surface near normal incidence appear curved outward, introducing a sub-millimeter to several-millimeter bias, so each radial measurement is first converted to camera-frame depth by

(1) $$Z=\frac{r}{\sqrt{1+x_{u}^{2}+y_{u}^{2}}}$$

where (*x*_*u*_, *y*_*u*_) are the undistorted normalized image coordinates obtained from the calibrated intrinsics. The pixel is then back-projected into the camera frame as *P* = *Z*(*x*_*u*_, *y*_*u*_ 1)^*T*^. This camera-frame point is rigidly transformed into the headset’s tracked local frame by the per-frame sensor pose, and into *F*_*ML*2_ by the SLAM-tracked world transform.

Absolute accuracy is evaluated against a matte white plane perpendicular to the optical axis at five standoffs {0.30, 0.40, 0.50, 0.60, 0.70} m. At each, 30 stationary frames are pooled, a best-fit plane recovered by orthogonal regression, and accuracy summarized by the mean, RMS, and 95th-percentile point-to-plane distance. Temporal consistency is evaluated separately: from 100 consecutive fixed-pose frames at 0.50 m, the temporal standard deviation of each pixel’s back-projected Z (valid in ≥ 80 % of frames) is computed and summarized by mean, median, and 95th percentile. Together these establish the admissible standoff range and the temporal noise floor that aggregation must average over.

### 2.3 RGB–D fusion and organ segmentation

The depth and RGB streams run concurrently: both begin at session start and stream uninterrupted. A scan is the synchronous latching of the latest depth and RGB frames at an operator-issued voice cue, rather than a sequential start–capture–stop transaction. This bounds the inter-stream temporal offset by per-frame jitter rather than warm-up time, essential for hand-held acquisition where any depth-to-RGB latency manifests as parallax in the colored cloud.

#### 2.3.1 Single-frame RGB–D fusion

**Fig. 1:**
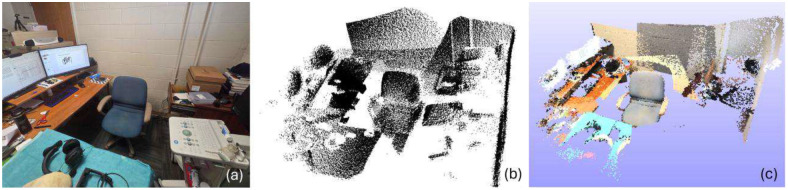
Magic Leap 2 depth-sensor and RGB–D fusion characterization in a laboratory environment. (a) Representative scene. (b) Multi-position depth point cloud from the onboard iToF sensor. (c) The same sweep colored by projection of each depth point through the calibrated RGB camera, illustrating that the two sensor frames register accurately across multiple acquisition poses, inheriting the RGB texture without visible parallax.

On each scan *i*, the latest flag-filtered, radial-to-Z-corrected depth cloud (in *F*_ML2_) is latched with the latest RGB frame, its pose, and the calibrated intrinsics. Each depth point is transformed into the RGB camera frame and projected by the pinhole-plus-distortion model, whose intrinsics are recovered by offline calibration; points behind the optical center or outside the image are culled, and each survivor takes the color of its nearest pixel. Each scan persists a pixel-to-3D correspondence table of (*u, v, p*_*w*_) for every retained point, with the raw image, intrinsics, and extrinsic. Any 2-D operation on the frame thus propagates to the 3-D cloud by mask lookup at (*u, v*) without re-projection. The raw images and extrinsics are the multi-view input for Method 2; the correspondence tables feed the segmentation below. Figure 1 shows a representative RGB–D acquisition in which the depth cloud, colored by projection through the calibrated RGB camera, registers coherently with no visible parallax, confirming the SLAM origin and calibrated sensor models are jointly accurate enough for quantitative reconstruction.

#### 2.3.2 SAM 2 integration

Both methods operate on the target organ only, whereas each frame also contains surrounding viscera, drapes, and instruments. Each frame is segmented offline with SAM 2 [10] in single-image promptable mode (SAM 2.1 Hiera-Large). Image-mode is used because the acquisitions are not regular-rate video but a small set of operator-cued snapshots at irregular intervals, across which video-mode temporal propagation is unreliable. The operator places positive-point prompts on visible organ and, where needed, negative prompts on adjacent structure to suppress mask leakage onto vessels, retractors, or drapes. SAM 2 runs in ambiguity-aware multi-mask mode, emitting three candidate masks with self-predicted IoU scores; the final mask is the argmax over those scores.

One convention must be reconciled: the ML2 SDK reports pixel coordinates with the origin at the bottom-left, whereas standard libraries use the top-left, so the v-axis of the correspondence table is flipped before any mask lookup, where *H* is the image height. The mask is then used in two ways: when paired with the depth correspondences, it defines a per-scan organ-only cloud, and when applied to the RGB stream, it restricts image-domain inference to the organ. Because the same masks drive both, the two reconstructions share an identical definition of organ pixels, eliminating one source of inter-method bias.

### 2.4 Surface reconstruction

**Fig. 2.**
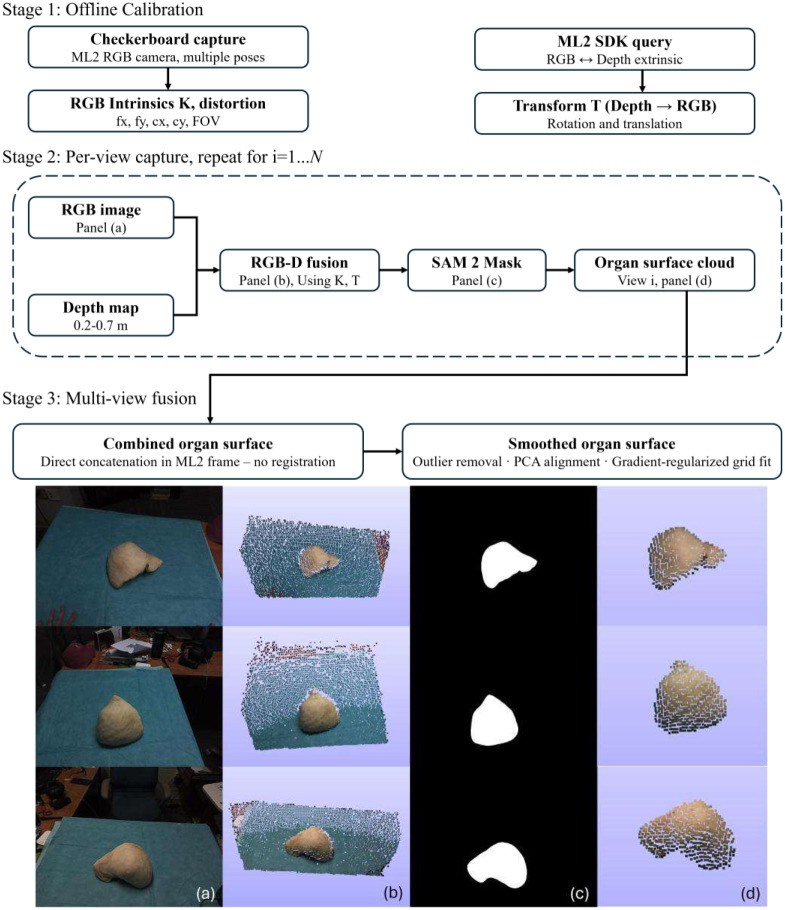
Sensor-based (Method 1) acquisition pipeline and representative per-view results. Top: pipeline schematic. Offline calibration of RGB intrinsics and the depth-to-RGB extrinsic; per-view RGB–D fusion, SAM 2 segmentation, and organ-only cloud extraction in *F*_ML2_; and multi-view fusion by direct concatenation (no inter-view registration) followed by outlier removal, PCA reorientation, and a gridded least-squares surface fit. Bottom (liver as illustrative example): three views from one sweep (a) raw RGB frame, (b) colored cloud, (c) per-frame SAM 2 mask, (d) organ-only cloud.

The end product is a measured three-dimensional organ surface in *F*_ML2_. Method 1 (Sec. 2.4.1) is obtained directly from the iToF stream and SLAM poses, giving a directly measured surface with no inter-view alignment. Method 2 (Sec. 2.4.2) ignores the iToF channel and recovers the surface from the RGB sweep alone using Depth Anything 3, anchored to reference scale by the SLAM-tracked camera centers. Their error profiles are complementary: the sensor-based surface is absolutely positioned but locally noisy and biased on translucent tissue, whereas the learning-based surface is locally smooth and free of iToF artifacts but carries a small residual offset along the camera optical axis, where parallax is weakest. Figure 2 places these per-view stages within the sensor-based pipeline.

#### 2.4.1 Sensor-based reconstruction (Method 1)

Let {*P*_Organ,i_} be the per-scan organ-only clouds from Sec. 2.3.2, each already expressed in *F*_ML2_ via the SLAM-tracked sensor pose and restricted to organ pixels by the per-frame mask. The Method 1 reconstruction is their union,

(2) $$P_{ML2}={⋃}_{i=1}^{N}P_{\text{Organ}}^{i}$$

with no inter-frame registration step, because all per-scan clouds are reported in the same world frame by the device’s SLAM reference, whose world-origin drift (Sec. 2.1) is comparable to the iToF sensor’s own absolute accuracy and therefore does not dominate the per-view error budget.

The aggregated cloud carries iToF-specific errors, including subsurface-scattering bias on translucent tissue, multi-path and flying-pixel artifacts at boundaries, faint banding, and a small surface-thickness from SLAM jitter. Rather than the raw union, it is post-processed into a smooth watertight surface to enable comparison with the learning-based surface, following the gridded surface-fitting of Collins et al. [11]. Statistical outlier removal discards points whose mean distance to their k = 15 nearest neighbors exceeds the cloud-wide mean by more than two standard deviations; the survivors are rotated by PCA so the minimum-variance direction is the height axis, and a smooth surface fit on a regular grid by a gradient-regularized least-squares system. Empty nodes are trimmed, surviving cells triangulated, and the mesh rotated back into *F*_ML2_. Because the fit penalizes only high-frequency departures, it removes the fringes, banding, and thickness while preserving the subsurface bias as a coherent displacement no iToF post-processing can remove which is precisely what motivates the learning-based reconstruction.

#### 2.4.2 Learning-based reconstruction (Method 2)

Because the iToF limitations are intrinsic to the measurement principle, a parallel branch ignores that channel and recovers organ geometry from the RGB stream using DA3 [8], a transformer-based geometric foundation model reported to outperform its predecessor for monocular depth and VGGT for multi-view depth and pose [8]. Because it relies solely on photometric correspondence across views, the method is by construction immune to subsurface-scattering and multi-path artifacts in the iToF pipeline. Its output is internally consistent but unitless; the ML2’s SLAM-tracked camera centers supply the measurement anchor.

The raw 1280 × 960 RGB frames for a sweep are passed to DA3 in one forward pass (DA3-Nested-Giant-Large-1.1, images-only mode). We bypass the offline RGB calibration here because DA3 estimates its own per-view intrinsics jointly with depth and pose, and pre-undistorting frames degraded the trajectory fit. For each view the network predicts a depth map, per-pixel confidence, extrinsic, and intrinsic in an arbitrary unitless frame *F*_DA3_. Because such networks produce occasional gross errors near specular highlights and occluding boundaries, each depth map passes through a global confidence gate dropping pixels below the q-th percentile of confidence pooled across the sweep, with q = 30 (q ∈ [20, 40] gave indistinguishable surfaces).

**Fig. 3.**
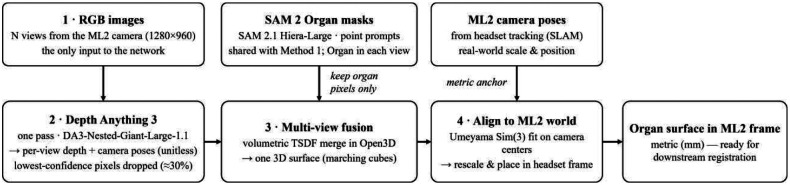
Learning-based (Method 2) reconstruction pipeline. The *N* RGB frames, the SAM 2 organ masks shared with Method 1, and the SLAM-tracked ML2 camera poses are the inputs. The frames are passed to Depth Anything 3, which predicts per-view depth and camera poses in a unitless frame; low-confidence pixels are dropped, the depths are fused by TSDF integration (Open3D) and masked to the organ, and the result is aligned to the ML2 world frame by a closed-form Umeyama Sim(3) fit on the camera centers, yielding a metric (mm) organ surface.

The confidence-masked depth maps are integrated into a truncated signed-distance (TSDF) volume in *F*_DA3_ [12], from which marching cubes extracts the zero-level surface; TSDF hyperparameters are set adaptively from the median valid depth to keep the voxel-to-scene-extent ratio roughly constant, with color integrated via Open3D [13]. To bring the geometry into *F*_ML2_, the DA3-predicted and SLAM-tracked camera trajectories, both synchronized by construction, provide *N* correspondences from which the similarity transform mapping *F*_DA3_ onto *F*_ML2_ is recovered by the closed-form Umeyama solution [14],

(3) $$\left(s^{*},R^{*},t^{*}\right)=\underset{s,R,t}{\text{arg}\;\text{min}}\;1/N\sum_{i}{\left\|sRp_{i}^{DA3}+t-p_{i}^{ML2}\right\|}^{2}$$

solved via the SVD of the trajectory cross-covariance, with the standard reflection correction so R* ∈ *SO*(3). The recovered similarity is applied to every vertex of the fused geometry, giving a metric reconstruction in *F*_ML2_. For the organ-only reconstruction, the SAM 2 masks are downsampled to DA3’s resolution and intersected with the confidence mask, and a second TSDF pass restricted to that mask yields the organ cloud, transported into *F*_ML2_ by the same transform, reusing the trajectory alignment rather than re-fitting on masked geometry avoids partial-overlap bias. Figure 3 summarizes the pipeline.

When sufficient viewpoints are available, Method 2 yields a clean, locally smooth organ cloud in *F*_ML2_ that avoids the subsurface and edge artifacts of Method 1. Its residual weakness is dependence on parallax, with errors most likely along the camera optical axis. The two surfaces thus trade absolute positional accuracy (Method 1) against local surface fidelity (Method 2); Sec. 2.5 quantifies this against ground truth.

### 2.5 Surface-reconstruction evaluation

The two reconstructions were evaluated on a phantom set designed to separate best-case accuracy from behavior under tissue-realistic optical conditions, since the iToF measurement depends on how the surface interacts with the near-infrared illumination. The set spans a range of surface-optical characteristics rather than one organ type: six objects were digitized against an independent ground truth including two anatomically distinct liver shapes, each cast in near-opaque white plaster and translucent red silicone (four liver phantoms), a surface-treated white silicone breast phantom, and an ex vivo porcine liver.

The phantom set was selected to isolate the effect of surface optics while preserving clinically relevant organ geometry. The core comparison used two liver shapes, each cast from the same mold in both near-opaque white plaster and translucent red silicone, so that geometry was held fixed while the interaction with the iToF illumination was varied. The plaster casts provide an opaque, diffuse best-case surface for active depth sensing, whereas the silicone casts reproduce the translucent soft-tissue condition in which subsurface light transport can bias iToF measurements. A surface-treated silicone breast phantom added a second anatomy and a strongly scattering skin-like surface, because mineral sunscreen contains titanium-dioxide and zinc-oxide particles that increase optical scattering and help emulate the near-surface scattering behavior of human skin [15, 16]. The ex vivo porcine liver provided the most tissue-realistic condition, including natural surface texture, absorption, specularity, and heterogeneity absent from cast phantoms.

**Fig. 4:**
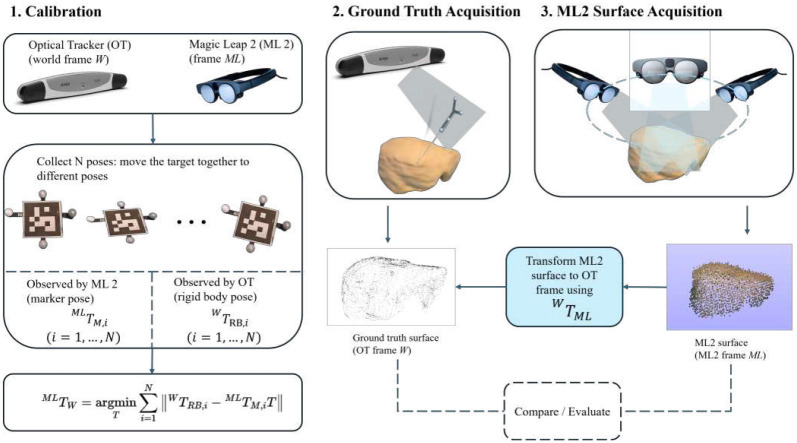
Surface-reconstruction evaluation workflow. (1) Hand-eye calibration co-registers the NDI Polaris Vega XT and the ML2 via a rigid body carrying retroreflective spheres and a planar ArUco marker. (2) A pivot-calibrated tracked stylus digitizes ground truth in *F*_OT_. (3) Each reconstruction is transported into *F*_OT_ and compared by directed point-to-point distance.

Each reconstruction was quantified against an independent, ground-truth surface acquired using a conventional optically tracked stylus (Fig. 4). Because the tracker reports coordinates in *F*_OT_ while the reconstructions live in *F*_ML2_, a one-time hand-eye calibration recovers the rigid transform *T*_OT→ML2_. Co-registration uses a custom rigid body exposing two co-located fiducial sets: four retroreflective spheres tracked by the NDI cameras and a planar ArUco board observed by the ML2 RGB camera. Across varied poses, the two sets satisfy an AX = YB relation; forming relative motions between pose pairs reduces it to the standard hand-eye problem, solved in closed form by the dual-quaternion method of Daniilidis [17]. The transform is held fixed and its residual on held-out poses reported as a calibration-quality metric.

Ground truth is digitized with a tracked pivot-calibrated stylus with residual pivot RMS below 0.5 mm for this work. For each phantom the operator sweeps the tip across the surface in a back-and-forth raster, transforming each position into *F*_ML2_; the accumulated set *P*_GT_ provides accurate metric ground truth but is spatially irregular, and is used as the basis to create a continuous smooth ground-truth surface by the same gridded surface-fitting used for the Method 1 cloud, so each reconstruction is compared against a dense reference rather than raw samples.

Each reconstruction is compared against *P*_GT_ by the directed point-to-point distance from each ground-truth point $p_{i}^{GT}$ to its nearest neighbor in the reconstruction,

(4) $$d_{i}=\underset{v\in P_{rec}}{\text{min}}\;\left\|p_{i}^{GT}-v\right\|$$

The ground-truth-to-reconstruction closest distance direction is preferred because the ground-truth points lie strictly on the physical surface whereas the reconstruction extends beyond the digitized region; the reverse would conflate reconstruction quality with sweep extent. We report two metrics: the RMSE of the directed distances as the primary measure, sensitive to the error tail, and the symmetric average closest-point distance (SACPD), the mean of bidirectional nearest-point distances within the common region of interest, so neither method is penalized for coverage outside the digitized region. The bidirectional SACPD additionally penalizes any surface extending beyond ground truth, guarding against hallucinated geometry.

Separately, the sensor-based cloud noise is characterized as the unsigned distance from every point of the unsmoothed iToF cloud to the fitted smoothed sensor surface of the same phantom. This residual quantifies the cloud thickness and outlier distribution that the smoothing removes, and is summarized by its mean, median, RMSE, 95th percentile, and maximum.

Each phantom was evaluated in five independent runs, each aggregating eight scans at 45° intervals around the object for a full angular sweep. The two ground-truth metrics and the raw sensor-cloud residuals are reported as mean ± SD across the n = 5 runs, the spread capturing run-to-run repeatability of the acquisition, segmentation, and reconstruction rather than specimen variability. Sensor-based and learning-based accuracy were compared with a two-way ANOVA (reconstruction method × material class), with the interaction as the primary test of the tissue-dependent crossover and Holm-corrected paired contrasts within each material; the two liver shapes provide within-class replication for the plaster and silicone conditions, whereas the breast and ex vivo classes comprise a single specimen each and their contrasts are correspondingly underpowered. Because the five runs are repeated acquisitions of the same physical object, the ANOVA residual is at the run level; the inference is therefore conditional on these six specimens and characterizes acquisition repeatability, not between-specimen variability. The interaction is effectively carried by the two replicated classes (plaster and silicone), each spanning two liver shapes, while the breast and ex vivo classes enter as single-specimen point estimates.

## 3 Results

### 3.1 Hologram stability

**Fig. 5:**
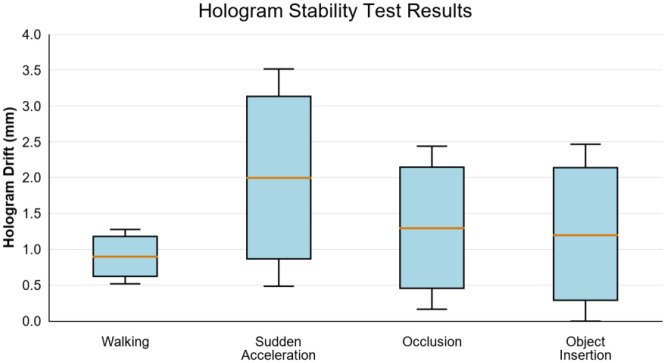
Hologram-stability results across the four operator-induced perturbations, as per-trial world-origin drift in the optical-tracker frame (*N* = 40 per condition). Boxes show the median and interquartile range; whiskers span the full range. All median drifts remain below 2 mm, with the largest spread under sudden head acceleration.

Across all four perturbation conditions the holographic world origin remained stable with median drift ≤ 2 mm for each condition (Fig. 5). Walking 7 m from the hologram and back gave the smallest, most tightly bounded drift (median 0.8 mm, upper whisker 1.3 mm). Sudden head acceleration was most demanding, with the highest median (2.0 mm) and widest spread (upper whisker 3.5 mm). Temporary sensor occlusion with re-localization (median 1.3 mm) and insertion of a new object (median 1.2 mm) gave comparable intermediate drift.

### 3.2 Depth-sensor accuracy and temporal consistency

**Fig. 6:**
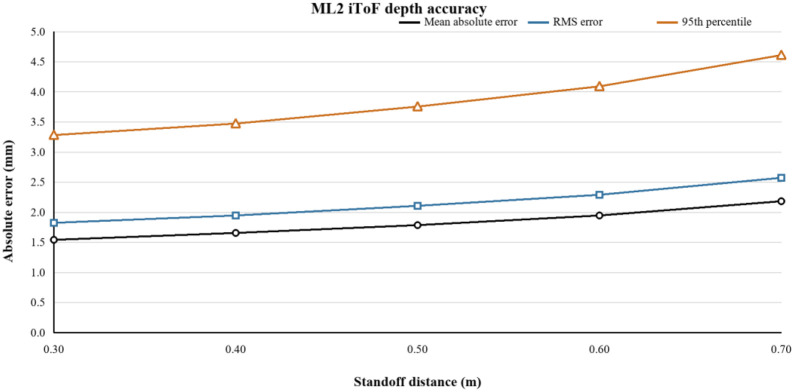
ML2 short-range iToF depth accuracy versus standoff. Mean absolute error (black), RMS error (blue), and 95th-percentile error (orange) of the point-to-plane distance to a matte white reference plane at five standoffs (0.30–0.70 m). All metrics increase smoothly with distance, with no abrupt degradation.

Across the tested 0.30–0.70 m working range, the ML2 short-range iToF measurements showed approximately 2 mm absolute accuracy. Mean absolute error increased modestly from 1.54 mm at 0.30 m to 2.18 mm at 0.70 m, and RMS error from 1.82 to 2.57 mm. The 95th-percentile absolute error stayed below 4.1 mm through 0.60 m and was 4.61 mm at the maximum standoff. A small positive range bias increased with distance, from 0.48 to 1.49 mm, with no abrupt degradation within the short-range interval (Table 1, Fig. 6).

**Fig. 7:**
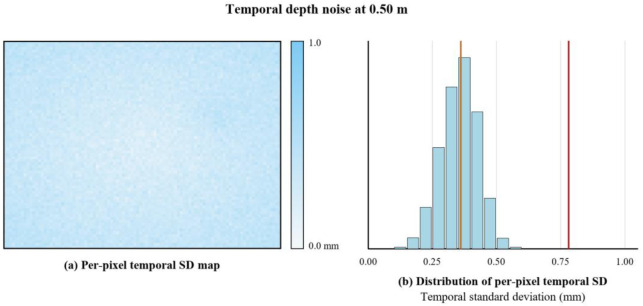
ML2 iToF temporal depth noise at 0.50 m, as the per-pixel temporal standard deviation of unprojected *Z* over 100 fixed-pose frames. (a) Spatial map across the field of view, showing low, uniform noise. (b) Distribution of per-pixel standard deviations; the orange line marks the median (0.36 mm), the red line the 95th percentile (0.78 mm).

At the representative 0.50 m standoff, 96.8 % of pixels remained valid in at least 80 % of the 100 frames. The per-pixel temporal standard deviation had a mean of 0.41 mm, a median of 0.36 mm, and a 95th percentile of 0.78 mm, substantially smaller than the approximately 2 mm absolute depth error (Fig. 7).

### 3.3 Surface reconstruction accuracy

**Fig. 8:**
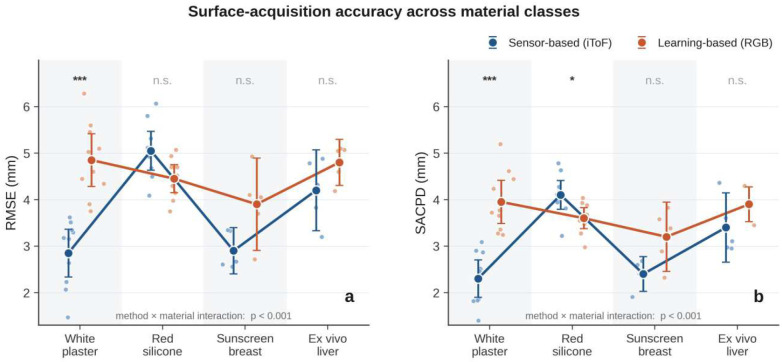
Surface-acquisition accuracy across material classes. Mean (a) RMSE and (b) SACPD for the sensor-based (iToF) and learning-based (RGB) reconstructions; points are individual runs, markers are means with 95% CI. The methods cross between opaque plaster and translucent silicone, a significant method × material interaction (p < 0.001, both metrics). Marks are Holm-corrected paired contrasts (***p < 0.001, *p < 0.05, n.s.); breast and ex vivo are single specimens (n = 5).

The final smoothed surfaces were evaluated against ground truth for all six phantoms, each value the mean ± SD across five runs (Table 2, Fig. 8). Accuracy splits by surface optics rather than organ shape. A two-way analysis (reconstruction method × material class) showed a significant method-by-material interaction (RMSE: F(3,52) = 14.3, p < 0.001, partial η^2^ = 0.45; SACPD: F(3,52) = 15.8, p < 0.001), consistent with a sensor-versus-learning ranking that depends on material for the specimens tested. Holm-corrected paired contrasts placed the sensor-based surface well ahead on opaque white plaster (2.0 mm lower RMSE, p < 0.001), with the ranking reversing on translucent red silicone (learning-based lower; significant on SACPD, p = 0.01, and at the nominal level on RMSE, p = 0.03). The sunscreen-coated breast and ex vivo porcine liver favored the sensor-based surface but did not reach significance, each being a single specimen (n = 5). Per-phantom values are given in Table 2, and SACPD followed the same ordering as RMSE.

**Fig. 9:**
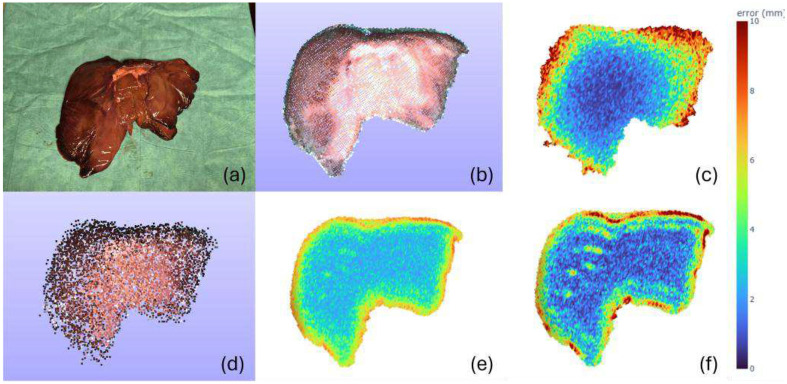
Surface reconstruction for the ex vivo porcine liver. (a) Photograph. (b) Learning-based (DA3) surface and (c) its per-point accuracy against ground truth. (d) Sensor-based (iToF) surface, (e) its raw per-point noise, and (f) its per-point accuracy against ground truth (sharing the color scale of c).

Raw sensor-cloud residuals to the fitted smoothed surface, as mean ± SD across five runs, are reported in Table 3. Residuals were smallest on the opaque white-plaster livers and largest on the translucent red-silicone phantoms, where subsurface scattering broadens the depth return; on every phantom the RMSE exceeded the mean, the signature of a right-skewed distribution dominated by a sparse high-magnitude tail. Per-point accuracy maps for the ex vivo porcine liver are shown in Fig. 9.

## 4 Discussion

The platform characterization establishes the ML2 as an admissible host for both paradigms. World-origin median drift stayed below 2 mm across all perturbations, and the worst-case whisker (3.5 mm, under sudden acceleration) is comparable to the sensor’s absolute depth accuracy (~2 mm) at an intraoperative standoff, and well below the centimeter-scale registration errors previously reported for onboard HMD depth sensing [7]. Because drift was measured in the independent frame *F*_OT_, these magnitudes reflect a genuine physical displacement of the hologram rather than a change internal to the headset. Over a single sweep, the world frame drifts by less than the random noise on an individual depth frame. Averaging many frames therefore suppresses the random per-view noise, while a small systematic range bias remains. Because the drift stays below the noise floor, the views from a sweep can be pooled directly into one frame without registering them to each other, which is the assumption both pipelines rely on.

The reported errors should be interpreted as surface-acquisition accuracy rather than final navigation accuracy. In deformable image-to-physical registration, the measured surface provides a dense intraoperative constraint on organ shape, not the surgical target itself. The 3–5 mm reconstruction errors observed on tissue-like surfaces, including 4.2 mm RMSE on the ex vivo porcine liver, therefore indicate a level of geometric fidelity relevant for deformation correction, albeit improvement is still needed. However, clinical adequacy ultimately depends on the downstream target-registration error after deformable registration, which should be evaluated in future intraoperative validation.

### 4.1 Sensor-based versus learning-based trade-offs

The central result is an accuracy that has a tissue-dependent optical property, tracking the optical interaction between surface and iToF illumination rather than organ geometry (Table 2). On the opaque white-plaster livers, where the return originates almost entirely at the geometric surface, the sensor-based reconstruction is more accurate by a clear margin (RMSE 2.6 ± 0.8 and 3.1 ± 0.6 mm versus 4.7 ± 0.6 and 5.0 ± 1.0 mm), reflecting a directly metric measurement needing no scale recovery. On the translucent red-silicone livers the ranking inverts: subsurface transport biases the iToF return toward the sensor and degrades its RMSE by roughly 2 mm per shape, while the learning-based RMSE is unchanged across materials. That both liver shapes, differing in curvature and aspect ratio, show the same crossover (Fig. 8), together with the significant method-by-material interaction, attributes the effect to surface optics rather than morphology.

The sensor-based reconstruction inherits iToF-specific errors that no per-pixel filter can remove. The most pronounced is subsurface scattering [18]: on translucent tissue some emitted photons return from subsurface scatterers rather than the geometric surface, biasing the measured distance toward the camera by an amount set by tissue optics; inherent rather than a rejectable failure mode, it survives both the quality flags and smoothing. Multi-path interference at boundaries produces aberrant pixels, of which the flags suppress the worst while a residual persists [19], alongside faint banding and surface-thickness from SLAM jitter. Because smoothing acts only on zero-mean high-frequency components, it removes these while preserving the subsurface bias as a coherent displacement. The raw residuals (Table 3) corroborate this: RMSE consistently exceeded the mean, indicating a right-skewed residual distribution. Thus, smoothing primarily removes a sparse population of large outliers, while leaving the coherent bias unchanged. This is why the learning-based branch, immune by construction, is the more accurate reference on translucent tissue.

The two extension phantoms sharpen this. On the sunscreen-coated breast phantom, whose titanium-dioxide and zinc-oxide loading drives scattering toward skin-like values and confines the return to a thin near-surface layer, the surface behaves as effectively opaque and the sensor-based method recovers its advantage (RMSE 2.9 ± 0.4 versus 3.9 ± 0.8 mm). The ex vivo porcine liver is the most informative case, combining genuine subsurface scattering with surface heterogeneity and specularity; here the sensor-based surface kept a modest advantage (4.2 ± 0.7 versus 4.8 ± 0.4 mm) rather than the silicone reversal, though within the run-to-run spread, suggesting the subsurface bias on real liver does not overturn the bulk accuracy of the directly-metric surface. The colormaps of Fig. 9 are consistent with this mechanism: the learning-based surface is locally smoother and avoids the sparse flying-pixel artifacts, although residual boundary-region discrepancies remain, likely reflecting weaker multi-view coverage and fusion constraints near silhouette regions [20]. The sensor-based surface, in contrast, retains lower bulk error across the interior. The silicone phantom thus isolates the subsurface mechanism as an adverse optical case, with the porcine liver between the extremes.

**Fig. 10.**
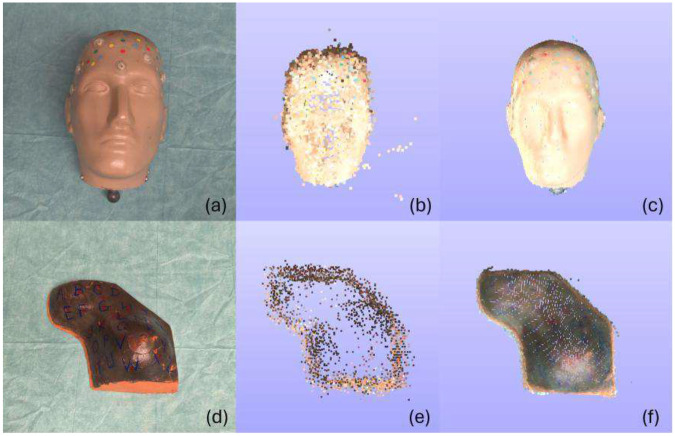
Qualitative reconstruction on two phantoms outside the iToF-cooperative regime. Top, a creased, specular face/head phantom: (a) photograph; (b) sensor-based surface, sparse and noisy from multi-path returns and specular saturation; (c) learning-based surface, recovering a coherent face from RGB alone. Bottom, a low-albedo black breast phantom: (d) photograph; (e) sensor-based surface, degraded by signal starvation; (f) learning-based surface, markedly cleaner.

Beyond subsurface scattering, two phantoms expose iToF failure modes unrelated to translucency. A creased, specular face/head phantom produces multi-path and flying-pixel returns at its depth discontinuities while its bright material saturates the sensor [21, 22], leaving the iToF cloud sparse (Fig. 10b); a low-albedo black breast phantom starves the active return and yields a degraded surface (Fig. 10e) [23]. In both, the RGB-only reconstruction recovers a usable surface (Fig. 10c, f) where the sensor pipeline degrades. Together with the silicone result, these cases show that the learning-based branch is robust to active-depth optical failure modes, including subsurface bias, specular saturation, and low-albedo starvation. The methods therefore occupy complementary regimes: sensor-based reconstruction is preferred when iToF returns are reliable, while learning-based reconstruction is useful when active depth is optically compromised and adequate viewpoints are available. These phantoms were qualitative and were not digitized against ground truth.

### 4.2 In vivo porcine demonstration

To test the pipeline outside the controlled setting, we ran it on an anaesthetized pig with the liver exposed on the operating bed. Two observations stand out. First, the sensor-based reconstruction was unexpectedly clean, with raw sensor-point residuals of mean 3.4 mm, median 3.1 mm, RMSE 4.2 mm, comparable to or below the ex vivo values of Table 3. A plausible explanation is the difference in surface state between the in vivo liver and the ex vivo liver. The in vivo liver retained an intact, moist serosal capsule under physiologic conditions, whereas the ex vivo liver had been excised, handled, stored, and sectioned before imaging. Ex vivo liver tissue can undergo rapid postmortem changes in biophysical properties within hours, including effects related to edema, vascular blood content, and later tissue degradation [24]. Dehydration also changes porcine liver optical properties, increasing absorption and effective attenuation with tissue water loss [25]. Because iToF depth depends on the phase and amplitude of actively returned near-infrared light, subsurface scattering can alter the measured optical path length and produce erroneous range values [18]. Therefore, the intact hydrated in vivo liver surface may have provided a stronger and more coherent first-surface return, while the ex vivo liver likely produced broader or less stable depth returns due to postmortem change, dehydration, and altered blood/surface condition. This remains a hypothesis because the two acquisitions differed in tissue handling, time postmortem, hydration, and imaging environment.

**Fig. 11.**
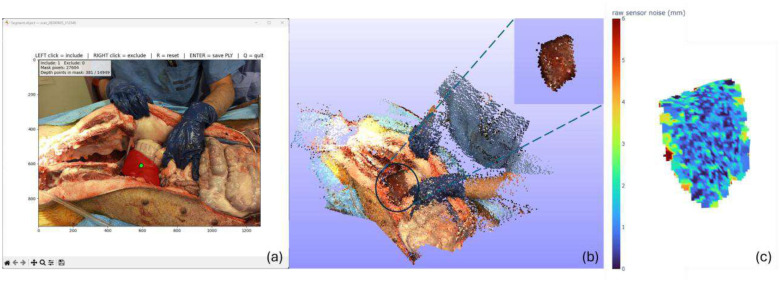
Intraoperative trial on an anaesthetized pig, liver exposed on the operating bed. (a) Interactive SAM 2 prompting through the ML2 RGB view, operator seed point on the liver. (b) Sensor-based (iToF) reconstruction within the surrounding scene, inset close-up of the segmented liver; the surface is notably low in noise. (c) Raw sensor-point noise colormap.

Second, the pig trial exposed the complementary limitation of the learning-based branch. Surgical access allowed the liver to be viewed mainly from one side, providing insufficient angular baseline for stable multi-view geometry and trajectory recovery. This is a structural requirement rather than a tuning failure: the RGB-only branch avoids active-depth optical errors but depends on viewpoint diversity, whereas the sensor branch can still produce a surface from a valid depth view. Thus, intraoperative practicality depends not only on optical surface properties but also on the viewpoints permitted by the surgical field. With no intraoperative ground truth, the result is qualitative (Fig. 11), the sensor-noise colormap serving as a consistency check on cloud thickness.

### 4.3 Limitations

Several limitations qualify these results. The segmentation stage is not yet real-time: each frame is segmented offline through operator-placed SAM 2 prompts, a manual step a live system cannot carry. All acquisitions used a fully exposed organ or phantom, free of the instruments, blood, smoke, and partial occlusion of an active field, under which robustness remains to be established. The comparison rests on five repeated acquisitions per phantom, characterizing run-to-run repeatability rather than specimen variability. The significance testing therefore treats material class as the factor with the two liver shapes as within-class replication; because the statistical error term is at the run level, the crossover is demonstrated within these specimens and is not, on this design, a specimen-level generalization; the breast and ex vivo classes each comprise a single specimen, so their contrasts are underpowered and should be read as indicative rather than confirmatory. The ground truth itself derives from stylus digitization that, though densified and surface-fit, is metrically authoritative only point-by-point, relying on the modality we motivate moving away from. Finally, the learning-based branch needs adequate multi-view coverage, which the pig trial showed cannot be assumed in a confined field.

## 5 Conclusion

We have presented a self-contained pipeline for intraoperative organ-surface acquisition, demonstrated on the liver, using a commercial MR headset and only the onboard sensors, and expressing every view in a single SLAM-tracked world frame with no need of inter-view registration. Platform characterization confirmed the ML2 world origin is stable to within the depth-sensor noise floor under realistic perturbations, and the short-range iToF sensor accurate to approximately 2 mm across the intraoperative standoff range, establishing the device as an admissible host for both paradigms.

A direct comparison against optically-tracked ground truth revealed a tissue-dependency on the optical characteristics of the surfaces scanned rather than organ geometry. The sensor-based reconstruction is the more accurate metric reference wherever the surface is cooperative to the iToF illumination such as opaque, diffuse or adequately bright, while the learning-based reconstruction is more robust on translucent, specular, and low-albedo surfaces, at the cost of a small residual position offset and a dependence on multi-view coverage. An in vivo porcine demonstration confirmed feasibility and exposed a practical asymmetry: under restricted single-side access the sensor-based surface degraded gracefully, whereas the multi-view reconstruction could not be formed.

Taken together, a commercial MR headset can serve as a self-contained intraoperative surface-acquisition device, eliminating the external hardware current practice requires, with the reconstruction paradigm selected according to the optical character of the target tissue and the viewpoint diversity the field permits. Integrating the acquired surface with downstream deformable image-to-physical registration and moving segmentation to a real-time, gaze-anchored prompt are the natural next steps toward a single-device MR navigation system.

## Figures and Tables

**Table 1.** Absolute depth-accuracy results (in mm).

Standoff (m)	Mean absolute error	RMS error	95th percentile	Signed bias
0.30	1.54	1.82	3.28	+0.48
0.40	1.65	1.94	3.47	+0.63
0.50	1.78	2.10	3.75	+0.86
0.60	1.94	2.29	4.09	+1.14
0.70	2.18	2.57	4.61	+1.49

**Table 2.** Accuracy of the final smoothed surfaces relative to ground truth, with per-material statistical contrasts (in mm).

*(a) Per-phantom accuracy, mean* ± *SD across five runs*.				
Phantom	Sensor RMSE	Learning RMSE	Sensor SACPD	Learning SACPD
Liver 1, white plaster	2.6 ± 0.8	4.7 ± 0.6	2.1 ± 0.6	3.8 ± 0.5
Liver 1, red silicone	4.8 ± 0.5	4.3 ± 0.5	3.9 ± 0.4	3.5 ± 0.4
Liver 2, white plaster	3.1 ± 0.6	5.0 ± 1.0	2.5 ± 0.5	4.1 ± 0.8
Liver 2, red silicone	5.3 ± 0.6	4.6 ± 0.3	4.3 ± 0.4	3.7 ± 0.2
Ex vivo porcine liver	4.2 ± 0.7	4.8 ± 0.4	3.4 ± 0.6	3.9 ± 0.3
Sunscreen breast	2.9 ± 0.4	3.9 ± 0.8	2.4 ± 0.3	3.2 ± 0.6

(b) Per-material paired contrasts (sensor vs learning), Holm-corrected across the four materials.					
Material	Sensor RMSE	Learning RMSE	Winner (RMSE)	RMSE p (Holm)	SACPD p (Holm)
White plaster	2.85	4.85	Sensor by 2.0 mm	< 0.001	< 0.001
Red silicone	5.05	4.45	Learning by 0.6 mm	0.083	0.014
Sunscreen breast	2.90	3.90	Sensor by 1.0 mm	0.25 (n.s.)	0.096 (n.s.)
Ex vivo liver	4.20	4.80	Sensor by 0.6 mm	0.27 (n.s.)	0.096 (n.s.)

Sensor and Learning columns in (b) are RMSE means pooled across each material’s phantoms and runs; the Winner column and RMSE p test the RMSE contrast, and SACPD p the corresponding SACPD contrast. A significant method × material interaction (two-way ANOVA, p < 0.001 for both metrics) is consistent with the tissue-dependent crossover; the ANOVA error term is at the run level, so this is established within these specimens rather than as a between-specimen generalization. The sunscreen breast and ex vivo liver classes each comprise a single specimen (n = 5).

**Table 3.** Raw sensor-point residuals to the fitted smoothed surface (in mm).

Phantom	Mean distance	RMSE
Liver 1, white plaster	2.0 ± 0.6	2.4 ± 0.7
Liver 1, red silicone	4.3 ± 1.2	5.0 ± 1.0
Liver 2, white plaster	2.3 ± 0.5	2.8 ± 0.6
Liver 2, red silicone	5.1 ± 0.8	5.8 ± 0.9
Ex vivo porcine liver	4.0 ± 1.1	4.7 ± 1.0
Breast phantom with sunscreen	2.7 ± 0.7	3.2 ± 0.8

## Data Availability

The data that support the findings of this study are available from the corresponding author upon reasonable request. The acquisition and reconstruction code is available from the corresponding author upon reasonable request.
